# Ten tips for utilizing AI to generate high quality OSCE stations in medical education

**DOI:** 10.3389/fmed.2025.1744657

**Published:** 2026-01-16

**Authors:** Imran Zafar, Hadeel Aboueisha, Ibrahim Elhassan, Fouzia Shersad, Suleyman Ayhan Caliskan, Asma Fatima Syeda, Mohammed Al-Houqani, Mohi Eldin Magzoub

**Affiliations:** 1 College of Medicine and Health Sciences, United Arab Emirates University, Al Ain, United Arab Emirates; 2 National Institute of Health Specialties, Al Ain, United Arab Emirates

**Keywords:** AI generated, assessment, guidelines, medical education, OSCE

## Abstract

Artificial intelligence (AI) is reshaping medical education, offering novel solutions to long-standing challenges in clinical assessment design. One of the most resource-intensive components of assessment is the development of high-quality Objective Structured Clinical Examination (OSCE) stations that are valid, reliable, and aligned with curricular outcomes. Traditional approaches to OSCE case creation are time-consuming, vulnerable to inconsistency, and difficult to scale. AI, particularly large language models, has emerged as a powerful tool for generating realistic, diverse, and customizable clinical scenarios. However, its safe and effective use in high-stakes examinations requires structured guidance and faculty oversight. This manuscript presents 10 evidence-informed, practical tips for leveraging AI to generate OSCE stations that are pedagogically sound and clinically authentic. These tips draw on focused literature review, experiential insights from AI-assisted case development, and consensus from medical education experts. Together, they provide a framework for integrating AI into assessment workflows while ensuring quality, fairness, security, and ethical compliance. By following these recommendations, educators and examination committees can enhance efficiency, maintain validity, and prepare learners for the complexities of modern clinical practice.

## Introduction

The landscape of medical education is rapidly evolving, driven by advances in technology, increasing expectations of healthcare systems, and the need to graduate highly competent clinicians (1). Clinical assessments, particularly the Objective Structured Clinical Examination (OSCE) and similar structured formats such as PACES (UK), SOE (Canada), and the Comprehensive Clinical Examination (CCE) used in the United Arab Emirates, remain the gold standard for evaluating a range of competencies, including history taking, physical examination, procedural skills, clinical reasoning, and communication (2).

Formats such as PACES, SOE, and CCE highlight the vast global variation in how clinical examinations are planned, scheduled, and finally graded. Though all of these formats share essential aims, there are significant variances in station length, assessment focus, examiner deployment, and standard-setting techniques (2, 3). This wide diversity presents a huge challenge for educational leaders, requiring them to regularly develop examples that are culturally appropriate, reproducible, and precisely linked to the assessment plan, often across various examination systems. To address this complexity, case development methodologies must be scalable and adaptive. AI-assisted technologies become particularly relevant in this context, with the ability to develop, alter, and standardize OSCE-type stations quickly across an array of different examination requirements.

It is precisely this growing complexity, driven by the diversity of OSCE formats, the expanding need for large examination banks across multiple specialties and assessment types, that highlights the need for more scalable and adaptive approaches to case creation. Under these conditions, the process of creating OSCE stations is labor-intensive and difficult to manage (3). Key challenges reported in the literature include ensuring blueprint coverage, achieving reproducibility, and maintaining fairness across stations and exam cycles (4). Inconsistencies in case quality, variability in examiner interpretation, and heterogeneous standardized patient (SP) portrayal can threaten validity and reliability (2). Moreover, traditional case-writing processes struggle to keep pace with rapidly evolving medical knowledge, emerging diseases, and updated clinical guidelines (5).

In this landscape, AI-assisted tools become particularly relevant, given their potential to generate, tailor, and standardize OSCE-type stations efficiently across a range of assessment formats. Artificial intelligence (AI) offers a transformative opportunity to address these challenges. Leveraging natural language processing, data synthesis, and pattern recognition, AI tools can generate realistic and diverse clinical scenarios, produce structured checklists, and rapidly adapt cases to specified learning outcomes (6). By integrating AI into the OSCE design process, educators can enhance scalability, reduce administrative burden, and support consistent quality across examination cycles (7).

Pilot studies report that AI-generated OSCE cases are feasible, useful, and time-saving, though expert oversight is required to ensure clinical accuracy (8). A randomized controlled trial confirmed that AI-generated practice stations are comparable to faculty-created ones in terms of student performance outcomes, suggesting their potential utility in formative and summative assessment preparation (9).

AI-powered virtual standardized patients (vSPs) further expand the potential of technology-enhanced assessment, allowing educators to simulate complex patient interactions and create highly realistic training encounters (10). These innovations collectively highlight the ability of AI to streamline case generation, enhance diversity and novelty in assessment content, and support scalability in high-stakes examinations.

Despite growing interest in using AI in assessment, existing frameworks remain broad and not tailored to the specific requirements of OSCE case development. Current recommendations is broadly focused on AI usage in education or assessment, but doesn't address critical OSCE-related requirements such as case authenticity, standardization, psychometric rigor, or safety safeguards. This gap underscores the need for a structured, evidence-informed framework to guide the responsible integration of AI into OSCE case creation. Moreover, uncritical use of generative AI can result in factual inaccuracies, algorithmic biases, and ethical risks. Human validation and structured oversight are necessary to ensure fairness and alignment with learning outcomes (11, 12).

To address this gap, we employed an exploratory qualitative methodology integrating expert consultation, iterative analysis and consensus-building to develop practical, evidence-informed recommendations.

This manuscript introduces 10 actionable tips for medical educators, program directors, and examination committees seeking to incorporate AI into OSCE station development. These recommendations are grounded in current literature (13), experiential evidence from implementation in a national clinical examination setting, and expert consensus. Collectively, they aim to help educators harness AI to create clinically authentic, psychometrically sound, and secure assessments that prepare learners for the realities of modern healthcare practice.

## Methods, setting, and context

### Study design

This work employed a qualitative, exploratory design to capture expert perspectives on the use of artificial intelligence (AI) for OSCE station generation. The approach focused on gathering experiential insights from educators who had directly implemented AI-assisted scenario development within high-stakes clinical examinations.

### Participants

Six medical education experts were recruited through purposive sampling, selected intentionally to ensure representation across undergraduate and postgraduate programs, clinical specialties, and assessment leadership roles. All participants had prior experience in OSCE design, blueprinting, and faculty development, and had also been involved in pilot projects that used AI tools for scenario generation.

### Data collection

Two members of the research team led an organized, in-person focus group meeting to gather data. In order to ensure that all participants responded to the same prompts and that fundamental topics were covered methodically, the session adhered to a set of pre-planned questions.

Due to the lack of audio recording, both facilitators took thorough notes in real time, recording participant reactions, areas of agreement or disagreement, and providing clarification on examples discussed. The facilitators checked and combined their notes right away to make sure they were accurate and comprehensive. Using standardized file-management practices, all data was safely kept on an encrypted institutional server.

Four predetermined domains were covered by the structured question set:

- Opportunities and challenges of using AI in OSCE case generation.
- Strategies for integrating AI into assessment workflows.
- Lessons learned from implementation during high-stakes examinations.
- Recommendations for faculty training, validation, and ethical safeguards.

The discussions were supplemented by the authors' experiential evidence from direct implementation of AI-assisted case generation during national OSCE clinical examinations.

### Setting and context

The study was conducted within the context of the National Institute of Health Specialties (NIHS), United Arab Emirates. NIHS is responsible for administering final written and clinical examinations for residency programs across multiple specialties. Since its establishment, NIHS has conducted more than 45 clinical examinations, engaging over 200 examiners from diverse disciplines and training backgrounds. The NIHS experience provided a robust, real-world perspective on integrating AI into high-stakes assessment design at scale.

### Data sources

Three distinct sources of information contributed to this work, each serving a different purpose and treated separately throughout the analysis:

1. Experiential evidence—This consisted of observational insights and reflective accounts from the authors' direct involvement in AI-assisted OSCE case development during national examinations. These data were not collected through the focus group but emerged from real-world implementation experiences.
2. Focus group notes—These notes were systematically documented during a structured, in-person expert focus group. They captured participant responses to predetermined questions, areas of agreement or divergence, and illustrative examples shared during the discussion. This dataset represents the formal qualitative evidence generated specifically for this study.
3. The focus group notes and experiential evidence were kept separate for analysis because they came from different places, had different goals, and had different levels of methodological control. Only the notes from the focus group were formally coded for themes. Experiential evidence was only used to put emerging themes in context and help us understand them, so that practice-based insights didn't change the way the coded dataset was set up.
4. Literature review—A focused review of peer-reviewed studies, conference proceedings, and scoping reviews published over the past 5 years on AI applications in medical education, clinical scenario generation, and assessment validity (14, 15).

### Data analysis

Braun and Clarke's reflexive thematic analysis framework, following the stages of familiarization, coding, theme development, and iterative refinement, was applied to synthesize findings from focus group discussions. The structured focus group notes were coded using an inductive method to make sure that themes were based on participant viewpoints rather than presumptions. Initial coding recommendations were produced by AI-assisted tools, but the research team manually reviewed, validated, and improved each code to guarantee accuracy, contextual sensitivity, and compliance with reflexive thematic analysis principles. Any disagreements were reviewed cooperatively and settled by team consensus. Emergent themes were organized into 10 concrete, evidence-informed recommendations. These recommendations were iteratively refined through author consensus meetings, incorporating illustrative examples from real examination scenarios to maximize relevance and applicability for educators.

## Results and literature review

### Findings from the literature

**Figure 1:**
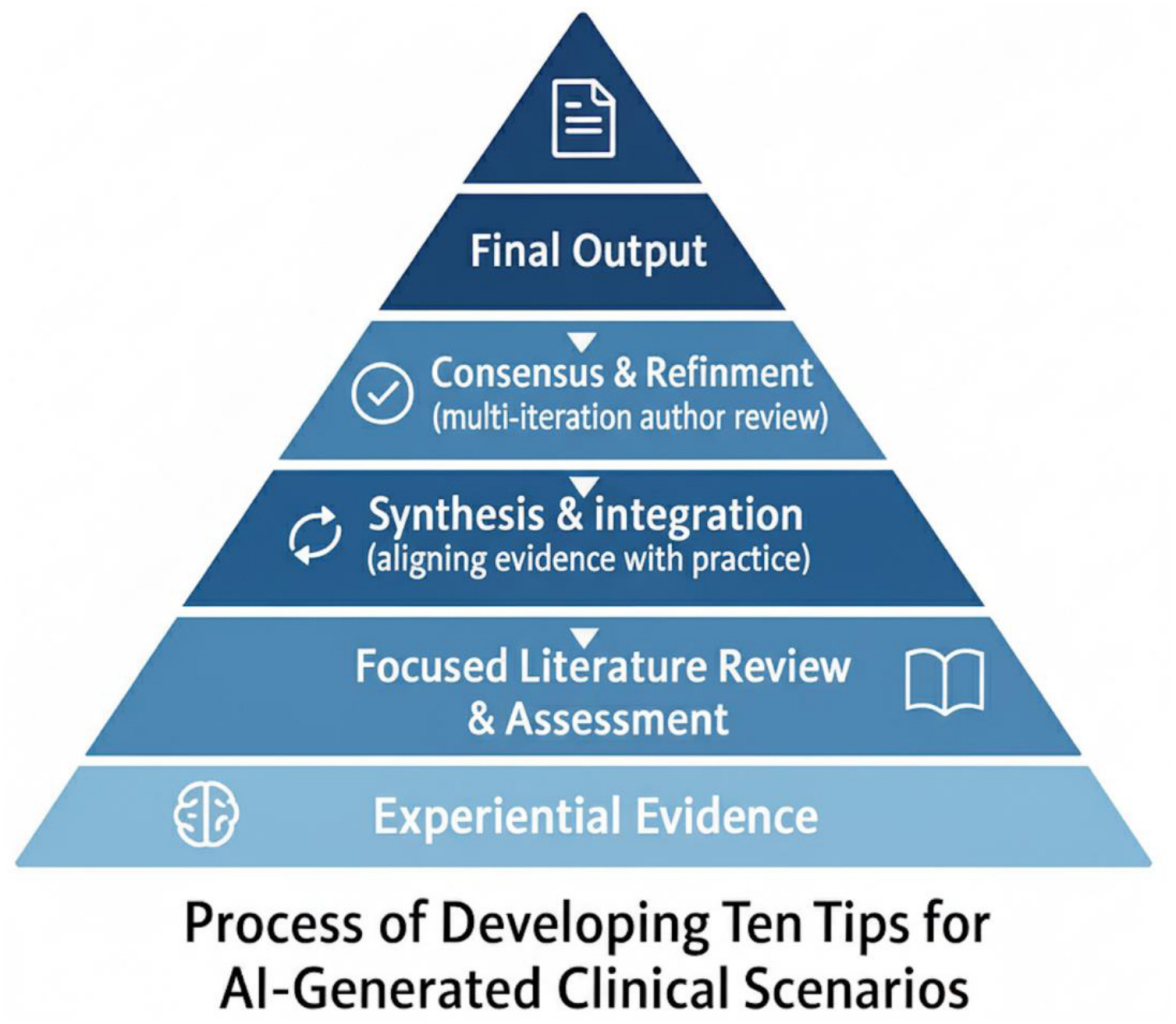
Developmental workflow for generating the 10 tips.

Findings from the focus group discussions, together with experiential evidence, highlighted key opportunities and challenges in the use of AI for OSCE development. These insights were complemented by a focused literature review, which revealed a rapidly expanding body of evidence on AI in medical education and assessment. Together, these sources formed the foundation of a broader developmental workflow in which evidence was synthesized and iteratively refined to generate the 10 practical tips presented in the Results, as illustrated in Figure 1.

Six key themes emerged:

1. AI for case and scenario generation

Large language models (LLMs) have shown the ability to generate realistic and diverse OSCE stations that mimic authentic patient presentations (16). Studies demonstrate that AI can produce structured checklists, candidate instructions, standardized patient (SP) scripts, and examiner probes with acceptable face and content validity (7, 10). However, faculty review is indispensable to ensure accuracy and curricular alignment (8).

2. Efficiency and scalability

AI-assisted case generation significantly reduces the time and effort required to produce examination materials (7). This efficiency enables broader coverage of clinical conditions, enhances sampling of competencies, and facilitates creation of large exam banks across multiple specialties (17).

3. Quality and validity considerations

Although promising, AI-generated outputs may contain factual inaccuracies, inconsistencies, or oversimplifications. Clinical, demographic, or cultural bias remains a concern. For these reasons, human oversight is essential to safeguard validity, reliability, and fairness (11).

4. Integration with assessment practices

Embedding AI-generated scenarios within programmatic assessment frameworks supports alignment with competency-based curricula and blueprint requirements (18). Emerging applications also include augmenting problem-based learning (PBL), adaptive testing, and blueprint design (19, 20).

5. Ethical and practical considerations

Key issues include data confidentiality, intellectual property rights, transparency, and risk of over-reliance on AI (11). Institutional policies, faculty training, and continuous QA processes are emphasized as critical enablers of safe use (15).

6. Practical implementation challenges and lessons learned

Implementing AI-assisted OSCE development presents several practical challenges. Institutional policies regarding data security, examination confidentiality, and approved digital tools may limit access to generative AI platforms, particularly for high-stakes assessments. Faculty preparedness varies widely, requiring targeted training in AI literacy, prompt design, and validation processes. Resource constraints, including limited access to secure enterprise-level AI systems and protected assessment management platforms, may further restrict implementation. During NIHS examinations, challenges included initial faculty skepticism, variability in prompt quality, and the need for additional review time to address AI-generated inconsistencies. Addressing these barriers requires institutional governance, phased implementation, and investment in faculty development and secure infrastructure.

## Future directions

Promising innovations include tailoring scenarios to Entrustable Professional Activities (EPAs), developing adaptive assessments, and leveraging multimodal AI (text, images, and audio) to create richer stations (15). Early work also highlights the role of AI in promoting equity by generating cases that reflect diverse patient populations (Table 1) (11).

**Table 1:** Literature themes and implications.

**Theme**	**Evidence from literature**	**Implications for clinical exams**
AI for case generation	LLMs generate OSCE stems, SP scripts, and checklists with acceptable validity (7, 10)	Can expand case diversity and authenticity if faculty oversight is applied. Thus, allowing for rapid production of clinically relevant scenarios yet requiring professional assessment to verify contextual accuracy and blueprint alignment.
Efficiency and scalability	AI reduces faculty workload and development time (7)	Enables larger exam banks and balanced blueprint coverage by supporting broader sampling of competencies and facilitating more frequent updates to assessment materials across specialties.
Quality and validity issues	Risk of factual errors and embedded bias (11, 15)	Requires SME validation, iterative refinement, and post-exam review to preserve psychometric defensibility, identify bias, and uphold fairness in high-stakes situations.
Integration with practice	Supports blueprinting, PBL, and adaptive testing (15)	Enhances curricular alignment and consistency by supporting standardized case structures throughout exam cycles and facilitating competency mapping at scale.
Ethical and practical issues	Risks of bias, security, and over-reliance (15)	Calls for institutional governance and training including faculty development, transparent usage guidelines, and safe AI platforms to protect test integrity.
Future directions	Adaptive, multimodal, and equity-focused AI (15)	Expands innovation potential in assessment design offering opportunities for more inclusive, dynamic, and authentic OSCE scenarios that reflect diverse patient populations.

**Figure 2:**
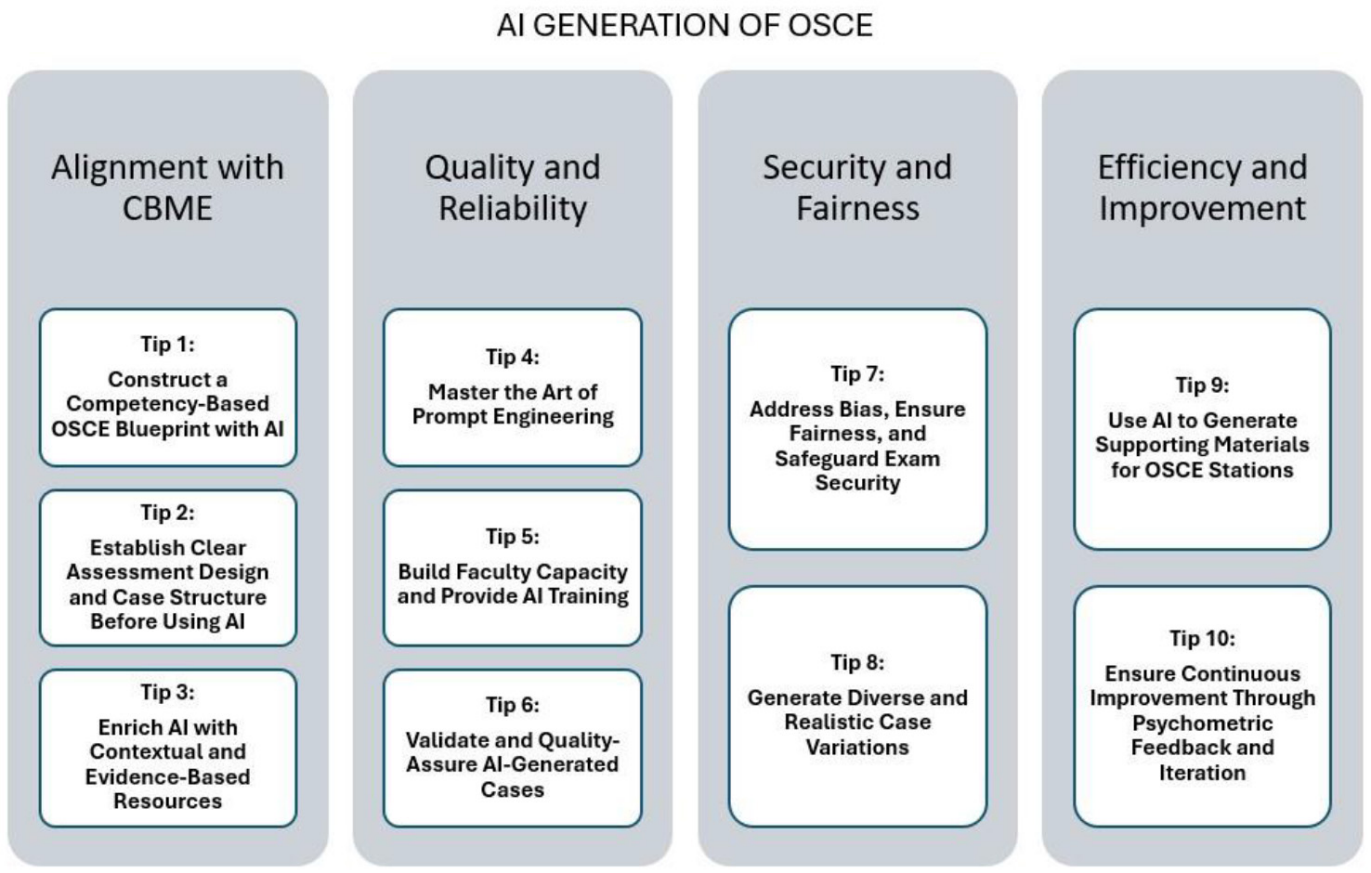
Conceptual framework for AI-supported OSCE station development.

To support conceptual integration of the findings, Figure 2 presents a visual synthesis of the 10 tips organized into four interrelated domains. This framework illustrates how AI-supported OSCE development spans competency alignment, quality assurance, fairness and security safeguards, and continuous improvement through feedback and psychometric monitoring. The figure serves as an orienting map for the detailed recommendations that follow.

### Tip 1: constructing a competency-based OSCE blueprint with AI

The development of a robust, competency-based blueprint is paramount to the quality and effectiveness of any Objective Structured Clinical Examination (OSCE).

#### Traditional blueprinting: processes and associated challenges

Historically, blueprinting establishes validity by systematically linking the examination's content to the corresponding curriculum objectives, clinical disciplines, and competencies. This methodology ensures the proportional representation of essential domains, including physical examination, clinical reasoning, communication, and history taking. However, the manual construction of these blueprints is significantly resource-intensive and often introduces inconsistencies, particularly when assessments span numerous specialties and utilize large question banks.

#### Leveraging AI for standa***r***dization and efficiency

AI presents a significant opportunity to standardize and substantially streamline the entire blueprinting workflow. By inputting program learning outcomes and established competency frameworks into the AI tools, the system can autonomously generate preliminary blueprint drafts that successfully align specific competencies, case types, and required skills with the appropriate level of training.

##### Example of a targeted prompt

A specific prompt, such as: “You are an OSCE assessment designer. Task: generate an OSCE blueprint for Year 3 undergraduate medical students, including the respiratory, cardiovascular, and gastrointestinal systems, ensuring alignment with the CanMEDS roles,” can quickly produce a comprehensive, structured draft for expert faculty review (Table 2).

**Table 2:** Blueprint snapshot (AI-assisted).

**Competency domain**	**# Stations**	**Level**	**Assessment focus**
History and communication	4	Year 3	Focused history + shared decision-making
Physical examination	3	Year 3	Cardiovascular and respiratory systems
Clinical reasoning	2	Year 3	Differential diagnosis and prioritization
Professionalism and ethics	1	Year 3	Informed consent and patient advocacy

This example shows how AI can operationalize curriculum outcomes into structured blueprints, ensuring proportional representation of competencies. To enhance novelty and avoid repetitive station designs, it is advisable to provide the AI system with examples of prior institutional blueprints. As a guiding principle, “*provide the AI system with examples of prior blueprints to avoid repetition and encourage novelty across exam cycles*.”

#### Key takeaway

AI can generate draft OSCE blueprints aligned with competencies and curricular outcomes, enhancing efficiency and coverage. However, expert review remains indispensable for ensuring contextual appropriateness and psychometric soundness.

### Tip 2: establish clear assessment design and case structure before using AI

AI is a powerful tool, but its outputs are only as effective as the inputs it receives. One of the most common pitfalls in using AI for OSCE case generation is providing vague prompts without a structured assessment framework. In the absence of clear guidance, AI may generate cases that are verbose, unfocused, or misaligned with curricular goals (20).

To mitigate this risk, faculty should define key assessment design parameters before engaging AI. Establishing these parameters provides the structural clarity necessary for AI systems to generate focused and relevant OSCE cases.

At a minimum, assessment designers should specify the intended station format, such as history taking, physical examination, data interpretation, counseling, or procedural skills. They should also identify the primary competency focus, for example communication, professionalism, or clinical reasoning, and clearly articulate the tasks expected of the candidate.

Timing constraints and scoring structure should be defined in advance. Typical timing ranges from 7 to 10 min for undergraduate OSCEs and 12–15 min for postgraduate assessments. Scoring approaches may include checklist based methods, global rating scales, or hybrid models. Together, these design decisions ensure that AI generated OSCE cases are aligned with assessment objectives and ready for faculty validation.

#### Structured prompting for targeted cases

Providing the AI with this explicit structure results in significantly more targeted outputs compared to generic requests.

##### Example of a structured prompt

Prompts that specify, “Create a 10-minute OSCE case for a Year 3 medical student on counseling a patient with new-onset type 2 diabetes, including candidate instructions, standardized patient role, and checklist with 10 items,” yield superior results compared to a vague request such as, “Write an OSCE case about diabetes.”

To facilitate this, institutions can develop case templates that standardize structure across specialties (21). These templates can then be embedded into AI prompts, reducing variability and enhancing reproducibility (Table 3).

**Table 3:** Structured case template.

**Section**	**Content**	**Notes**
Candidate Instructions	“You are the junior doctor in the clinic…”	Clear, time-limited
Patient (SP) role	45-year-old patient with chest pain	Include demeanor, key phrases
Candidate tasks	Take focused history, counsel patient on results	Align with competency framework
Scoring checklist	12 items, each mapped to skill domain	Include global rating component

#### Key takeaway

AI performs best when given structured guidance. Pre-defining station format, competencies, and scoring structure ensures that AI-generated OSCE cases are aligned, targeted, and ready for faculty validation.

### Tip 3: enrich AI with contextual and evidence-based resources

The quality of AI generated OSCE cases depends strongly on the specificity and richness of the contextual information provided in the prompt. When prompts lack sufficient clinical, institutional, or cultural context, AI systems tend to produce generic or poorly contextualized scenarios that may not align with local practice or learner needs (8).

Enriching prompts with references to clinical guidelines, institutional assessment policies, and local epidemiological patterns strengthens content validity and improves alignment with authentic clinical practice.

This approach is particularly important for assessment because AI can be deliberately guided using established evidence based frameworks such as Entrustable Professional Activities, CanMEDS roles, or EmiratesMEDs competencies. Anchoring prompts to these frameworks helps ensure that generated cases target clearly defined learning outcomes and assessment purposes (18, 22). Embedding these frameworks into prompts improves consistency across specialties and supports competency-based medical education (CBME).

In practical terms, prompt enrichment may include explicit reference to relevant clinical guidelines such as those issued by the ADA, NICE, or WHO, incorporation of demographic and cultural characteristics that reflect local patient populations, and alignment with institutional or national competency frameworks such as EmiratesMEDs, CanMEDS, or ACGME. Specifying disease prevalence or priority conditions based on local health needs further enhances relevance and educational defensibility.

The contrast between a weak and an enriched prompt illustrates this effect clearly. A prompt such as “Write an OSCE case on hypertension” is likely to yield a broad and unspecific scenario. In contrast, a prompt that asks the AI to “Generate a 10 min OSCE case for a Year 4 medical student in the UAE, focusing on counseling a patient recently diagnosed with type 2 diabetes, aligned with ADA 2025 guidelines, and reflecting cultural considerations in diet and lifestyle” produces a more realistic, contextually grounded, and assessment ready case.

#### Key takeaway

Enriching AI prompts with guidelines, cultural context, and competency frameworks ensures that OSCE cases are not only clinically accurate but also relevant, socially accountable, and aligned with curricular outcomes.

### Tip 4: master the art of prompt engineering

The effectiveness of AI in OSCE case generation depends largely on the quality of the prompt provided. Poorly constructed prompts often yield vague, overly simplistic, or irrelevant scenarios (8). Conversely, structured, precise, and layered prompts can generate highly relevant cases, complete with candidate tasks, standardized patient (SP) roles, and scoring checklists (7).

*Prompt engineering* is, therefore, a critical skill for educators using AI in assessment design. This involves breaking down requests into clear instructions, embedding contextual details, and specifying the desired output format. For example, prompts should identify the clinical scenario, learner level, competencies targeted, case duration, and assessment format. Adding output constraints (e.g., number of checklist items) supports standardization and enhances the defensibility of OSCE scores (20). Importantly, educators can also ask the AI itself to refine or recreate a better prompt, allowing for iterative improvement and more tailored case outputs.

Well engineered prompts play a critical role in ensuring that AI generated assessment materials accurately reflect the intended competencies and assessment constructs. By explicitly defining the task, target learner level, clinical focus, and competency framework, structured prompting reduces the risk of construct irrelevant variance and minimizes the introduction of unintended difficulty or bias. This is particularly important in OSCE design, where small variations in case framing can substantially influence performance.

In addition, structured prompting supports reproducibility and consistency across examination cycles. When prompts are standardized and systematically documented, AI generated cases can be regenerated, reviewed, and refined in a controlled manner, supporting fairness, comparability, and quality assurance within the assessment process.

A weak prompt such as “Write an OSCE case about asthma” provides minimal guidance to the AI and is therefore likely to generate a broad, generic scenario with limited educational or assessment value. In contrast, a well structured prompt that specifies the learner level, clinical context, task duration, and required outputs leads to a far more targeted and usable case. For example, asking the AI to “Generate a 10 min OSCE case for Year 4 undergraduate students on the acute management of an adult with asthma exacerbation in the emergency department, including candidate instructions, a standardized patient script with key phrases, examiner prompts, and a 12 item checklist mapped to communication, history taking, and clinical reasoning domains” results in a coherent, assessment ready OSCE station that is clearly aligned with intended competencies and scoring criteria.

The second prompt provides specificity (clinical scenario, learner level, setting, task) and constraints (duration, checklist length, competency mapping), resulting in a structured and usable output that supports standardization and inter-rater reliability.

Effective prompt engineering benefits from a structured and sequential approach. Using stepwise instructions, such as requesting the AI to first generate the case stem, followed by the standardized patient role and then the scoring checklist, helps control output flow and improves internal consistency.

Explicitly specifying competency alignment further strengthens prompt quality. Referencing established frameworks such as CanMEDS roles or EmiratesMEDs competencies ensures that AI-generated cases are clearly linked to intended learning outcomes and assessment standards.

Providing clear output constraints is also essential. Defining parameters such as the number of checklist items, station duration, and expected level of detail helps standardize AI outputs and reduces unnecessary variation across cases.

Prompt development should be treated as an iterative process. Refining prompts based on initial AI outputs, using structured feedback loops, allows educators to progressively improve relevance, clarity, and alignment with assessment goals.

Finally, employing meta-prompts, such as asking the AI to assess a prompt for clarity, completeness, or alignment with objectives, can further enhance design quality. This reflective layer supports more deliberate and defensible use of AI in OSCE case development.

Educators should also recognize that prompt refinement is iterative: initial outputs often require follow-up instructions such as “revise checklist to reduce redundancy” or “adjust SP role to reflect cultural communication styles.” This iterative dialogue ensures accuracy and contextual fit (19). Iteration also reduces construct drift and helps maintain fidelity to assessment specifications.

#### Key takeaway

Prompt engineering transforms AI from a “generic text generator” into a structured assistant for OSCE design. Faculty who masters this skill can unlock AI's potential for creating clinically authentic, competency-aligned, and exam-ready cases.

### Tip 5: build faculty capacity and provide AI training

AI can enhance OSCE case development, but its outputs are not self-validating. Faculty play a central role in ensuring accuracy, fairness, and alignment with curricular outcomes (18). Across the literature, recurring limitations; such as factual inaccuracies, cultural or demographic bias, and inconsistency in output detail, were identified, reinforcing the need for purposeful faculty preparation (8). Therefore, capacity building and faculty development are essential for safe and effective integration of AI into assessment design. Strengthening faculty capability also supports defensible assessment decisions and reduces variability in how AI tools are used across departments.

#### Core competencies for AI-enabled assessment design

Faculty development in AI enabled assessment design should focus on building a coherent set of competencies rather than isolated technical skills. Educators need a clear understanding of what AI systems can and cannot reliably do in assessment contexts, including commonly reported limitations and failure modes documented in the literature. This foundational knowledge allows faculty to use AI as a supportive tool rather than an uncritical content generator.

Equally important are prompt engineering skills that support reproducible and purpose driven case generation. Faculty should be able to design prompts that specify learner level, assessment intent, competency targets, and expected outputs, thereby improving consistency and usability across assessment cycles. Training should also address validation and quality assurance practices, enabling educators to systematically review AI generated content for factual accuracy, bias, and alignment with intended constructs.

Ethical considerations must be embedded throughout this training, including issues related to fairness, equity, data security, and the protection of examination confidentiality. Finally, faculty should be familiar with institutional guidelines governing the responsible use of AI in assessment to ensure consistency, transparency, and appropriate oversight across programs and departments.

Workshops and short training modules can build these skills effectively (Table 4). Embedding AI training into faculty development programs ensures long-term sustainability (13). Early adopters can support institutional rollout by mentoring colleagues and participating in communities of practice that encourage shared learning and reduce duplication of effort (15).

**Table 4:** Mini-curriculum for AI training in assessment.

**Module**	**Learning objectives**	**Format**
Introduction to AI in assessment	Explain capabilities, limitations, and use cases	Seminar + demo
Prompt engineering	design structured prompts for OSCE case generation	Hands-on workshop
Validation and QA	Apply frameworks for reviewing AI-generated cases	Peer-review session
Ethics and fairness	Identify risks of bias, inequity, and exam security issues	Case discussion
Practical application	Generate and refine an OSCE case with AI support	Simulation exercise

#### Key takeaway

Faculty development is the cornerstone of safe AI integration. Training educators in AI literacy, prompt design, and validation methods empowers them to harness AI responsibly, ensuring assessment quality and fairness.

### Tip 6: validate and quality-assure AI-generated cases

Even when prompts are carefully structured, AI generated OSCE cases may still contain factual inaccuracies, cultural mismatches, or internal inconsistencies. If these issues are not systematically identified and corrected, they can undermine exam validity, fairness, and reliability (23). For this reason, AI generated cases should not be adopted directly for formative or summative assessment without a clearly defined validation and quality assurance process.

Validation should begin with expert review, in which subject matter experts examine AI outputs for clinical accuracy, alignment with current guidelines, and relevance to the local clinical and educational context (21). This step is essential for identifying subtle clinical errors or inappropriate assumptions that may not be immediately apparent in automated outputs.

Following expert review, cases should be cross checked against the assessment blueprint to confirm that they address the intended competencies and are appropriately positioned within the overall examination structure (3). A subsequent peer review by an independent faculty member can further strengthen quality by identifying gaps, redundancies, or inconsistencies in task design, scoring criteria, or case flow.

Pilot testing provides an additional safeguard by exposing the case to a small group of students or residents prior to formal use. This step helps identify ambiguities, timing problems, and imbalances within checklists or examiner prompts, which are particularly common in early AI generated drafts that vary in depth, pacing, and clarity (24). After implementation, ongoing psychometric monitoring is required to evaluate case performance using indicators such as difficulty indices, discrimination, and inter rater reliability, thereby confirming that the case functions as intended in practice (9).

Although this process closely resembles traditional quality assurance procedures for OSCE development, it must be deliberately adapted to address AI specific risks, including hallucinated content and embedded biases. Systematic validation therefore remains a critical safeguard when integrating AI generated cases into assessment programs (Table 5).

**Table 5:** AI case validation workflow.

**Step**	**Process**	**Outcome**
AI case generation	Draft OSCE case using structured prompt	Baseline scenario created
SME review	Expert checks for accuracy and guideline adherence	Errors corrected, context aligned
Peer review	Second reviewer evaluates structure and checklist	Standardization and refinement
Pilot testing	Small-scale trial with learners/SPs	Identifies timing or clarity issues
Psychometric analysis	Post-exam metrics reviewed	Confirms validity and reliability

This streamlined workflow illustrates how AI output integrates into existing QA systems while requiring adaptations for AI-specific risks.

#### Key takeaway

AI can accelerate case creation but cannot replace human oversight. A structured validation workflow ensures that AI-generated OSCE stations are accurate, fair, and psychometrically robust.

### Tip 7: address bias, ensure fairness, and safeguard exam security

AI systems learn from large datasets that often embed historical, cultural, or clinical biases. Left unchecked, these biases can appear in OSCE cases, perpetuating stereotypes or underrepresenting important patient groups (23). For example, AI may default to male patients for cardiac cases or neglect culturally sensitive expressions of symptoms (25). Such biases undermine fairness and may disadvantage certain candidate groups.

Additionally, AI introduces new exam security risks. Cases generated or stored on unsecured platforms could be accessed by candidates, raising concerns about content leakage and academic integrity (26). Institutions must therefore combine bias detection with robust security protocols.

Promoting fairness and safeguarding exam security are critical when integrating AI into OSCE development. One essential step is systematic bias screening, which helps counteract AI's tendency to reproduce default demographic patterns or generate unrepresentative cases. Faculty should ensure diversity across patient demographics, including age, gender, ethnicity, and socioeconomic status, avoid stereotypical portrayals such as linking specific conditions or behaviors to particular groups, and include clinical conditions that reflect local epidemiology.

Fairness should also be evaluated through structured audits of AI-generated cases. Mapping cases to blueprint domains helps ensure balanced representation across competencies and content areas, while review by subject matter expert panels allows identification of unintended differences in case difficulty across learner subgroups.

Cultural adaptation is another key consideration. Standardized patient scripts should be reviewed and adapted to reflect locally appropriate communication styles and sociocultural norms. Involving regional educators in reviewing language, cues, and contextual details helps ensure authenticity and reduces the risk of cultural mismatch.

Robust security measures are necessary to protect exam integrity. Institutions should use secure, institutional, or encrypted AI platforms and avoid public tools for generating or storing final examination content. AI-generated cases should be housed within secure assessment management systems that maintain access logs, and case variants should be rotated across exam cycles to reduce predictability and content leakage.

Finally, fairness and security require ongoing monitoring after exam administration. Performance data should be analyzed across variables such as gender, language, or educational background to detect differential item functioning. Stations that demonstrate evidence of unfairness or bias should be revised or retired to maintain the integrity and defensibility of the assessment.

#### Example—NIHS case review

During an NIHS OSCE cycle, an AI-generated communication case described a single-parent household where the mother was assumed to be the caregiver. Faculty reviewers flagged this as gender-stereotyped and revised the case to make the caregiver role neutral. Subsequent psychometric analysis showed no gender-based performance gap, supporting fairness.

This example demonstrates how fairness checks and psychometric monitoring intersect in evaluating AI-generated content.

#### Key takeaway

Bias and security are non-negotiable in AI-assisted assessment. Institutions should adopt a bias audit checklist, cultural review, and secure storage protocols to ensure fairness, protect exam integrity, and maintain public trust in high-stakes assessments.

### Tip 8: generate diverse and realistic case variations

Reusing identical OSCE stations across exam cycles increases the risk of content leakage and predictability. This undermines fairness by rewarding candidates who have prior access to cases, rather than those demonstrating competence (3). Traditionally, creating multiple case versions is resource-intensive, limiting the number of variations available for high-stakes exams.

AI can generate multiple permutations of a core case by varying demographics, context, or complicating factors while preserving the intended learning outcomes (27). This not only strengthens exam security but also improves authenticity by reflecting the variability seen in real-world clinical practice (9).

Variation generation also helps mitigate recurring AI limitations; such as overly generic outputs, by prompting more contextualized and diverse scenarios from a single validated core case.

The generation of multiple case variants using AI should begin with clearly defining the core elements that must remain constant across all versions. These include the primary diagnosis, the intended competencies to be assessed, and the essential checklist items that underpin scoring and decision making. Fixing these elements helps ensure construct consistency and protects against unintended shifts in difficulty.

Once the core elements are established, selected variables can be deliberately modified to create meaningful variation. These may include patient demographics such as age, gender, or ethnicity, as well as differences in clinical context such as emergency, outpatient, or rural settings. Additional variation can be introduced through comorbidities, including the presence or absence of relevant risk factors, and psychosocial factors such as caregiver involvement or language barriers, provided these do not alter the underlying construct being assessed.

Structured prompts should then be used to instruct the AI to generate a limited number of variants, typically three to five, while explicitly maintaining comparable difficulty, scope, and station timing. Clear constraints within the prompt are essential to prevent drift in case complexity or assessment focus across versions.

Each generated version must undergo independent validation to confirm clinical accuracy, fairness, and alignment with the assessment blueprint, following the same quality assurance principles applied to single AI generated cases. Finally, validated case variants can be rotated across examination circuits or assessment sessions to reduce recall bias and enhance exam security, while preserving comparability across cohorts (Table 6).

**Table 6:** Case variations for acute chest pain.

**Version**	**Demographics**	**Context**	**Key variation**
A	58M, smoker, diabetic	ED after exercise	Classic ACS with risk factors
B	45F, no comorbidities	At rest after emotional stress	Differentiate ACS vs. panic attack
C	70M, hypertensive	Mild confusion with dyspnea	Geriatric considerations
D	52F, postpartum	Pain radiating to back	Include PE as differential

This approach ensures that all versions test the same learning outcomes (history, risk factor identification, urgent management) but in diverse and realistic contexts.

#### Key takeaway

AI-generated case permutations enhance exam security, broaden content diversity, and mirror clinical variability. Faculty should carefully define fixed vs. flexible elements, validate each version, and strategically rotate them across exam cycles.

When managed systematically, variation generation also reduces AI's tendency toward repetition and ensures more equitable, realistic, and defensible assessments.

### Tip 9: use AI to generate supporting materials for OSCE stations

Developing an OSCE case requires much more than writing a clinical vignette. Each station needs candidate instructions, standardized patient (SP) scripts, examiner checklists, global rating scales, and sometimes supplementary resources such as lab results, imaging, or patient education materials. Traditionally, creating these materials is highly time-consuming and requires coordination among multiple faculty (27).

AI can streamline this process by generating structured supporting materials from a validated case stem. This not only reduces faculty workload but also enhances consistency across exam circuits, improving fairness and reproducibility (7).

#### Steps to generate supporting materials with AI

The development of supporting materials using AI should begin only after a case has been fully validated through expert review and blueprint alignment, as outlined in Tip 6. Using unvalidated cases as inputs risks propagating clinical inaccuracies or construct misalignment across all associated materials. A validated core case provides a stable foundation for generating standardized patient instructions, examiner tools, and supplementary resources.

Standardized patient scripts can then be generated to support consistent portrayal across candidates. These scripts should explicitly describe the patient's affect, such as appearing anxious or distressed, and clearly specify verbal cues, including spontaneous complaints and information that should be disclosed only if directly elicited. Non verbal guidance, such as facial expressions, posture, or body language, should also be included. Particular emphasis should be placed on clarity and precision, as vague or overly narrative AI generated scripts are prone to misinterpretation by standardized patients.

Examiner tools should be created in parallel to ensure alignment with the intended assessment constructs. AI can be prompted to generate structured checklists with anchored scoring categories, such as 0 to 2 or 0 to 3 scales, alongside global rating scales that capture overall clinical reasoning and communication skills (24). Anchored scoring frameworks are especially important for supporting inter rater reliability and limiting variability introduced by loosely structured AI outputs.

AI may also be used to generate supplementary materials, including laboratory results, ECG summaries, imaging descriptions, or patient information leaflets for communication focused stations. These materials require careful review, as AI generated clinical data may contain internal inconsistencies or values that do not align with the clinical narrative of the case.

Finally, all supporting materials should be reviewed collectively to ensure internal consistency. Candidate instructions, standardized patient scripts, examiner checklists, and supplementary data must align in terms of clinical details, timing, and expected performance to preserve fairness and assessment validity.

An example of an AI generated standardized patient script excerpt illustrates this approach. The patient is described as appearing anxious and frequently holding the chest, with an opening line stating that the pain started 45 min ago. If asked, the patient reports nausea and sweating while denying cough, and non verbal behavior includes grimacing and pressing a hand against the sternum. Such structured detail supports consistent portrayal and clearer interpretation by standardized patients (Table 7).

**Table 7:** Checklist (excerpt).

**Item**	**Score (0–2)**
Asked onset, duration, character of pain	0–2
Asked about radiation (jaw, arm, back)	0–2
Inquired about cardiovascular risk factors	0–2
Suggested urgent ECG	0–2

Global rating scale:

1 = unsatisfactory: missed key data, poor organization
2 = borderline: partial data, limited prioritization
3 = satisfactory: systematic, identified ACS features
4 = excellent: comprehensive, prioritized urgent action

#### Key takeaway

AI can generate SP scripts, examiner guides, and supplementary data from a single validated case, saving time and ensuring consistency. Faculty review remains essential to ensure clinical accuracy and cultural appropriateness.

### Tip 10: ensure continuous improvement through psychometric feedback and iteration

Introducing AI into OSCE case generation is not a one-time innovation but a continuous process. Even well-designed, validated stations require ongoing evaluation to confirm that they function as intended in real testing environments. Psychometric feedback and iterative refinement ensure that AI-generated cases remain valid, reliable, and fair over time (3).

This ongoing evaluation is particularly important because several limitations identified across the literature; such as inconsistency in output structure, variable difficulty, and occasional factual errors, can only be detected through post-administration monitoring.

#### Steps for continuous improvement

Continuous improvement of AI generated OSCE stations should be grounded in systematic post examination evaluation. After each assessment cycle, key psychometric metrics should be collected, including item difficulty based on score distributions, discrimination indices that indicate how well the station differentiates between higher and lower performing candidates, and inter rater reliability across examiners (24). Together, these indicators help determine whether AI generated stations perform comparably to traditionally developed ones and support defensible assessment decisions.

Quantitative data should be complemented by structured feedback from candidates, examiners, and standardized patients. Candidate feedback is particularly useful for identifying issues related to clarity of instructions and perceived fairness, while examiner and standardized patient feedback can reveal ambiguities, unrealistic portrayals, or practical challenges in case execution that may not be evident from psychometric data alone.

Based on these findings, cases should be revised iteratively. This may involve refining checklists, clarifying instructions, or adjusting station timing to better match the intended level of performance. AI can be used efficiently at this stage to regenerate revised versions, provided that the validated core content and competency targets are retained. Such iterative revision helps mitigate construct drift and ensures that the station continues to assess the intended competencies across multiple examination cycles.

Maintaining a clear audit trail is an essential component of this process. Records of revisions, associated psychometric results, and the rationale for changes support transparency, accountability, and institutional learning. Finally, insights gained through this continuous improvement cycle should be integrated into faculty development activities. Sharing psychometric findings and practical lessons in training workshops can strengthen prompt design skills and enhance faculty capacity for critical review of AI generated assessment materials.

##### Example—Iterative refinement at NIHS

During an NIHS exam cycle, an AI-generated chest pain case initially showed poor discrimination (index = 0.18). Post-exam review revealed redundant checklist items and an overly generous marking scheme. Faculty revised the station, simplifying the checklist and clarifying SP cues. In the next cycle, the case achieved a discrimination index of 0.36, meeting accepted psychometric standards. This example demonstrates how psychometric monitoring can diagnose AI-related design issues and guide targeted refinement.

#### Key takeaway

AI-assisted OSCE case generation should be treated as an iterative cycle rather than a static process. Systematic use of psychometric data and user feedback ensures continuous improvement, supporting long-term validity, reliability, and fairness.

## Discussion

This paper provides 10 evidence-informed recommendations for the responsible integration of artificial intelligence (AI) into the development of Objective Structured Clinical Examination (OSCE) stations. Together, these tips address the dual goals of efficiency and quality assurance, recognizing that AI can accelerate case generation but requires structured oversight to maintain validity, reliability, and fairness.

AI's capacity to generate diverse, structured, and customizable scenarios has the potential to reduce faculty workload and improve blueprint coverage (7, 13). However, its outputs are not self-validating and may include factual inaccuracies, biases, or cultural mismatches (8, 23). The recommendations presented here highlights how to collectively provide a coherent strategy for integrating AI tools into assessment workflows while preserving essential human oversight.

### Alignment with competency-based assessment

Tips 1–3 highlight the importance of blueprinting, structured case design, and contextual enrichment. The broader implication is that AI enhances competency-based assessment only when its use is anchored in explicit curricular expectations and contextualized with local clinical realities. AI does not inherently ensure competency alignment; this alignment is created through deliberate educational design choices embedded within prompt structure and case specifications (21).

### Ensuring quality and reliability

Tips 4–6 focus on prompt engineering, faculty training, and validation processes, which are critical to mitigating the risks of flawed or biased outputs. The validation workflow, which combines subject matter expert (SME) review, blueprint cross-checks, peer feedback, and psychometric monitoring, parallels best practices in traditional OSCE design while adapting them to the AI era (3). This iterative, multi-step validation ensures stations are not only clinically accurate but also psychometrically robust.

This suggests a hybrid quality model in which human expertise and psychometric evidence jointly regulate the reliability of AI-assisted assessments.

### Safeguarding fairness and security

Tips 7 and 8 emphasize equity and exam security, highlighting the need for bias audits, cultural adaptation, and the use of diverse case permutations. AI can unintentionally reproduce stereotypes or exclude minority patient profiles (25); deliberate diversity in AI prompts, combined with fairness audits and post-exam analysis for differential item functioning, helps safeguard equity. Generating case variations also mitigates the risks of item leakage, strengthening the integrity of high-stakes exams (27).

When these findings are interpreted widely, fairness and security appear not as discrete tasks, but as systemic safeguards required for justifiable AI usage in high-stakes assessments. AI prompts must actively counteract demographic defaults, and case modification methodologies achieve two goals: increasing authenticity while decreasing predictability and item exposure.

### Enhancing efficiency and authenticity

Tips 9 and 10 illustrate how AI can streamline the generation of supporting materials (SP scripts, examiner checklists, multimedia resources) and facilitate continuous improvement through psychometric feedback. These innovations reduce administrative burden, improve consistency across circuits, and foster authenticity by mirroring real-world variability in clinical encounters (24). Beyond efficiency, an important implication is that AI generated materials, when validated, can enrich authenticity by offering dynamic variations and multimodal resources that more closely mirror real clinical environments. Moreover, integrating post exam psychometric data into AI assisted revision cycles reflects a shift toward continuous quality enhancement rather than one time case design.

### Implications for future research

Despite early support for the feasibility of AI assisted OSCE generation (9, 18), findings from focus group discussions revealed notable skepticism and highlighted several critical areas requiring further investigation. Participants expressed particular concern about the reliability and psychometric defensibility of AI generated materials, reflecting uncertainty about whether such outputs can consistently meet established assessment standards.

One key priority identified was the need for rigorous validation of psychometric properties, including reliability and generalizability, across diverse educational and clinical contexts. Addressing these concerns is essential to demonstrate consistency of output and to strengthen evidence for content validity. Participants also emphasized the importance of examining whether AI generated case variations are effective in maintaining exam security, as predictability and item leakage were perceived as ongoing challenges despite the use of multiple case versions.

Further inquiry was recommended into the applicability of AI generated assessments beyond undergraduate medical education. Participants questioned whether similar approaches could be extended to interprofessional and competency based assessments across other health professions, raising issues of generalizability and contextual transferability. In addition, the long term impact of faculty development initiatives emerged as an important area for research, particularly in light of the wide variation in confidence, readiness, and risk awareness observed among faculty users.

Collectively, addressing these research gaps will help establish a stronger empirical foundation for institutional policy development and accreditation guidance, ensuring that the adoption of AI in assessment is aligned with principles of quality, fairness, and equity.

### Limitations of the developmental workflow

While the developmental workflow efficiently combined experiential evidence, focus group insights, and expert consensus, these sources inevitably had limits. Experiential perspectives are frequently linked to local practices and institutional cultures, thereby limiting the applicability of findings to other educational settings. Furthermore, expert agreement is inevitably influenced by the opinions and assumptions of participating educators, notably their expectations about AI readiness or established evaluation standards. Variations in participants' prior experience with AI technologies may possibly have altered the prioritizing of perceived difficulties or opportunities. These limits do not diminish the validity of the recommendations, but rather highlight the need for ongoing empirical validation across a wide range of programs and situations.

## Conclusion

Artificial intelligence (AI) is reshaping clinical assessment design by offering innovative solutions to long-standing challenges in OSCE development. Evidence from recent reviews and guidance indicates growing feasibility and utility of AI tools when applied within competency-based assessment frameworks (13, 18).

This paper provides a structured, evidence-informed framework of Ten Tips to guide safe integration of AI into high-stakes examinations. By leveraging AI for blueprinting, case generation, supporting materials, and case variations, assessment teams can increase efficiency, expand content diversity, and enhance authenticity (9, 24, 27). At the same time, structured validation, faculty training, bias audits, and psychometric monitoring are essential safeguards to ensure validity, reliability, fairness, and exam security (3, 23).

AI should serve as a complement, not a replacement, to faculty expertise. Successful implementation balances the speed and scalability of AI with human judgment, cultural sensitivity, and ethical oversight (18).

Future work should evaluate psychometric outcomes of AI-generated stations across contexts, examine security benefits of case permutations, and develop governance models that align innovation with accountability (9, 13, 23). Responsible adoption offers a pathway to more efficient, equitable, and authentic assessments that better prepare learners for modern clinical practice.

## References

[1] Frenk J Chen L Bhutta ZA Cohen J Crisp N Evans T . Health professionals for a new century: transforming education to strengthen health systems in an interdependent world. Lancet. (2010) 376:1923–58. doi: 10.1016/S0140-6736(10)61854-5 21112623

[2] Khan KZ Gaunt K Ramachandran S Pushkar P. The objective structured clinical examination (OSCE): AMEE guide no. 81. Part II: organisation & administration. Med Teach. (2013) 35:e1447–63. doi: 10.3109/0142159X.2013.818635 23968324

[3] Pell G Fuller R Homer M Roberts T. How to measure the quality of the OSCE: a review of metrics – AMEE guide no. 49. Med Teach. 32, 802–811. doi: 10.3109/0142159X.2010.507716 20854155

[4] Nyangeni T ten Ham-Baloyi W van Rooyen DRM. Strengthening the planning and design of objective structured clinical examinations. Health SA. (2024) 29:2693. doi: 10.4102/hsag.v29i0.2693 39229317 PMC11369580

[5] Berbenyuk A Powell L Zary N. Feasibility and educational value of clinical cases generated using large language models. Stud Health Technol Inform. (2024) 316:1524–8. doi: 10.3233/SHTI240705 39176494

[6] Khakpaki A. Advancements in artificial intelligence transforming medical education: a comprehensive overview. Med Educ Online. (2025) 30:2542807. doi: 10.1080/10872981.2025.2542807 40798935 PMC12351741

[7] Misra SM Suresh S. Artificial intelligence and objective structured clinical examinations: using ChatGPT to revolutionize clinical skills assessment in medical education. J Med Educ Curric Dev. (2024) 11:23821205241263475. doi: 10.1177/23821205241263475 39070287 PMC11273588

[8] Taylor-Drigo CA Kumar A. Strengths and limitations of using ChatGPT: a preliminary examination of generative AI in medical education. medRxiv. (2025). doi: 10.1101/2025.03.12.25323842

[9] Ali M Rehman S Cheema E. Impact of generative AI on the academic performance and test anxiety of pharmacy students in OSCE: a randomized controlled trial. Research Square. (2024). doi: 10.21203/rs.3.rs-5283600/v1 39903358

[10] Öncü S Torun F Ülkü HH. AI-powered standardised patients: evaluating ChatGPT-4o's impact on clinical case management in intern physicians. BMC Med Educ. (2025) 25:278. doi: 10.1186/s12909-025-06877-6 39979969 PMC11843762

[11] García-López IM Trujillo-Liñán L. Ethical and regulatory challenges of generative AI in education: a systematic review. Front Educ. (2025) 10:1565938. doi: 10.3389/feduc.2025.1565938

[12] Winkler PW Zsidai B Hamrin Senorski E Pruneski JA Hirschmann MT Ley C . A practical guide to the implementation of AI in orthopaedic research—Part 7: risks, limitations, safety and verification of medical AI systems. J Exp Orthop. (2025) 12:e70247. doi: 10.1002/jeo2.70247 40276496 PMC12019299

[13] Rincón EHH Jimenez D Aguilar LAC Flórez JMP Tapia ÁER Peñuela CLJ. Mapping the use of artificial intelligence in medical education: a scoping review. BMC Med Educ. (2025) 25:526. doi: 10.1186/s12909-025-07089-8 40221725 PMC11993958

[14] Amiri H Peiravi S Rezazadeh Shojaee SS Rouhparvarzamin M Nateghi MN Etemadi MH . Medical, dental, and nursing students' attitudes and knowledge towards artificial intelligence: a systematic review and meta-analysis. BMC Med Educ. (2024). 24:412. doi: 10.1186/s12909-024-05406-1 38622577 PMC11017500

[15] Gordon M Daniel M Ajiboye A Uraiby H Xu NY Bartlett R . A scoping review of artificial intelligence in medical education: BEME Guide No. 84. Med Teach. (2024) 46:446–70. doi: 10.1080/0142159X.2024.2314198 38423127

[16] Park Y-J Guo E Sachdeva M Ma B Mirali S Rankin B . OSCEai dermatology: augmenting dermatologic medical education with Large Language Model GPT-4. Can Med Educ J. (2025) 16:29–31. doi: 10.36834/cmej.80056 41584944 PMC12826809

[17] Mishra GV Luharia AA Naqvi W Sood A. Artificial intelligence in OSCE: innovations and implications for medical education assessment – a systematic review. In: 2024 2nd DMIHER International Conference on Artificial Intelligence in Healthcare, Education and Industry (IDICAIEI) (Wardha). (2024). p. 1–5. doi: 10.1109/IDICAIEI61867.2024.10842789

[18] Masters K MacNeil H Benjamin J Carver T Nemethy K Valanci-Aroesty S . Artificial intelligence in health professions education assessment: AMEE Guide No. 178. Med Teach. (2025) 47:1410–24. doi: 10.1080/0142159X.2024.2445037 39787028

[19] Heston TF. Prompt engineering for students of medicine and their teachers. arXiv [preprint]. (2023). arXiv:2308.11628. doi: 10.48550/arXiv.2308.11628

[20] Zaghir J Naguib M Bjelogrlic M Névéol A Tannier X Lovis C. Prompt engineering paradigms for medical applications: scoping review and recommendations for better practices. J Med Internet Res. (2024) 26:e60501. doi: 10.2196/60501 39255030 PMC11422740

[21] Daniels VJ Pugh D. Twelve tips for developing an OSCE that measures what you want. Med Teach. (2018) 40:1208–13. doi: 10.1080/0142159X.2017.1390214 29069965

[22] ten Cate O Chen HC Hoff RG Peters H Bok H van der Schaaf M. Curriculum development for the workplace using Entrustable Professional Activities (EPAs): AMEE Guide No. 99. Med Teach. (2015) 37:983–1002. doi: 10.3109/0142159X.2015.1060308 26172347

[23] Amann J Blasimme A Vayena E Frey D Madai VI. Explainability for artificial intelligence in healthcare: a multidisciplinary perspective. BMC Med Inform Decis Mak. (2020) 20:310. doi: 10.1186/s12911-020-01332-6 33256715 PMC7706019

[24] Hodges B Regehr G McNaughton N Tiberius R Hanson M. OSCE checklists do not capture increasing levels of expertise. Acad Med. (1999) 74:1129–34. doi: 10.1097/00001888-199910000-00017 10536636

[25] Pupic N Ghaffari-zadeh A Hu R Singla R Darras K Karwowska A . An evidence-based approach to artificial intelligence education for medical students: a systematic review. PLoS Digit Health. (2023) 2:e0000255. doi: 10.1371/journal.pdig.0000255 38011214 PMC10681314

[26] Burstein J LaFlair GT. Where assessment validation and responsible AI meet. arXiv [preprint]. (2024). arXiv:2411.02577. doi: 10.48550/arXiv.2411.02577

[27] Lam G Shammoon Y Coulson A Lalloo F Maini A Amin A . Utility of large language models for creating clinical assessment items. Med Teach. (2025) 47:878–82. doi: 10.1080/0142159X.2024.2382860 39186054

